# Correction: Silica based ZnFe_2_O_4_ nanocomposite as a novel photocatalyst for basic fuchsin dye degradation

**DOI:** 10.1038/s41598-026-47898-5

**Published:** 2026-04-21

**Authors:** Mohamed M. Desouky, Mamdouh El-Sayed, Ahmed M. El-Khawaga

**Affiliations:** 1 Nanoscience and Technology Program, Faculty of Science, Galala University, Galala City, New Galala City, Suez, 43511 Egypt; 2 Department of Basic Medical Sciences, Faculty of Medicine, Galala University, Galala City, Suez, 43511 Egypt

Correction to: *Scientific Reports* 10.1038/s41598-026-41259-y, published online 20 March 2026

In the original version of the Article, Figure 9 was a duplicated version of Figure 10.

The correct Figure 9 is now updated in the original article.

The original Article has been corrected.

